# Cerebral Arterial Air Embolism Associated with Mechanical Ventilation and Deep Tracheal Aspiration

**DOI:** 10.1155/2012/416360

**Published:** 2012-08-01

**Authors:** S. Gursoy, C. Duger, K. Kaygusuz, I. Ozdemir Kol, B. Gurelik, C. Mimaroglu

**Affiliations:** Department of Anesthesiology, School of Medicine, Cumhuriyet University, 58140 Sivas, Turkey

## Abstract

Arterial air embolism associated with pulmonary barotrauma has been considered a rare but a well-known complication of mechanical ventilation. A 65-year-old man, who had subarachnoid hemorrhage with Glasgow coma scale of 8, was admitted to intensive care unit and ventilated with the help of mechanical ventilator. Due to the excessive secretions, deep tracheal aspirations were made frequently. GCS decreased from 8–10 to 4-5, and the patient was reevaluated with cranial CT scan. In CT scan, air embolism was detected in the cerebral arteries. The patient deteriorated and spontaneous respiratory activity lost just after the CT investigation. Thirty minutes later cardiac arrest appeared. Despite the resuscitation, the patient died. We suggest that pneumonia and frequent tracheal aspirations are predisposing factors for cerebral vascular air embolism.

## 1. Introduction

Embolism of air into the cerebral vessels is an iatrogenic complication of numerous invasive medical procedures performed in anesthesia and intensive care [1] and may occur either in artery or in vein if an entry in the arterial or venous circulation is created [2–6]. Arterial air embolism associated with pulmonary barotrauma has been considered a rare, but a well-known complication of mechanical ventilation [7]. The volume of intravascular air embolised in the cases is usually small. Massive air embolism to the major cerebral vessels is extremely rare. We report a patient with an unusual air collection at the cerebrum during positive pressure ventilation. 

## 2. Case

**Figure 1:**
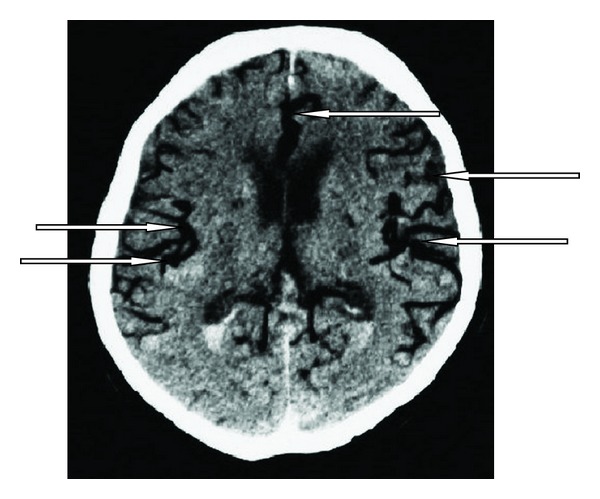
A section of cranial CT scan in the 8th day of admittance. Arrows show arterial air embolism.

**Figure 2:**
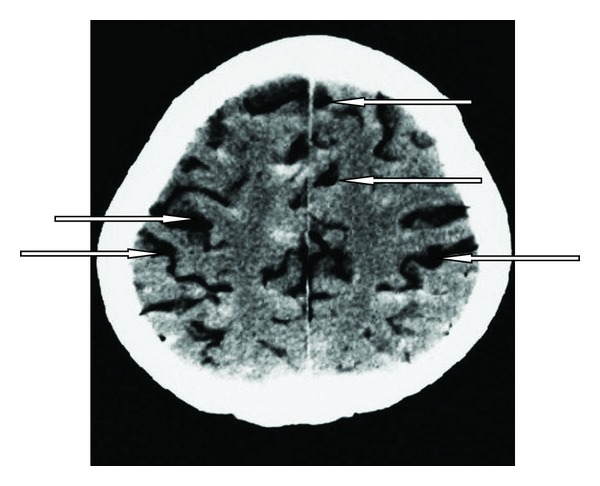
A section of cranial CT scan in the 8th day of admittance. Arrows show arterial air embolism.

A 65-year-old man known with long-lasting arterial hypertension arrived at the emergency department in a subcomatose state. Subarachnoid hemorrhage (SAH) was seen on CT scan of brain. When admitting to intensive care unit, Glasgow coma scale (GCS) of the patient was 8 and he had spontaneous but insufficient breathing. Thus he required intubation after 1 hour of ICU admission. The patient was ventilated by mechanical ventilator in spontaneous mode. Four days after intubation the patient had excessive bronchial secretions and nosocomial pneumonia was diagnosed. Due to the excessive secretions, deep tracheal aspirations were made frequently. GCS was 8–10, and he was breathing in spontaneous mode in mechanical ventilator. GCS decreased from 8–10 to 4-5, and the patient was reevaluated with cranial CT scan in the 8th day of admittance. In CT scan, air embolism was detected in the cerebral arteries (Figures 1 and 2). The patient deteriorated and lost spontaneous respiratory activity just after the CT investigation. The airway pressure increased and pulmonary compliance decreased. Arterial blood pressure and saturation of the arterial oxygen decreased and cyanosis appeared. Meanwhile, GCS was 3. Treatment was started for the air embolism but 30 minutes later cardiac arrest occurred. Despite the resuscitation, the patient died. 

## 3. Discussion

Cerebral vascular air embolism may be seen either in artery or in vein [8]. Cerebral air embolism most commonly results from arterial air embolism [9]. On the other hand, retrograde rising of air bubbles in the venous system has been demonstrated in an experimental setting [10]. The complication of cerebral vascular air embolism may result either severe neurological injury or death. The arterial embolism has higher mortality than the venous air embolism; the latter may has a good prognosis. The air may be absorbed spontaneously in venous embolisms. In mechanic ventilation with positive pressure, barotraumas may cause the air embolism [8, 11, 12]. There is a predisposition for the mechanical ventilation induced air embolism, in the patients with thorax trauma, pneumothorax and bronchoscopy administration [11–13]. Symptoms vary with the location of the occlusion and the size of the air bubbles [10]. Hemoptysis and sudden cardiac and cerebral dysfunction of the mechanically ventilated patients should suggest the cerebral air embolism [14]. In our case, sudden cerebral dysfunction was observed. Continuous positive pressure ventilation was a predisposing factor for pulmonary infection and pulmonary infection induced excessive pulmonary secretions caused frequent tracheal aspirations. 

In conclusion, we suggest that pneumonia and frequent tracheal aspirations are predisposing factors for cerebral vascular air embolism which should not be ignored in mechanically ventilated patients.
